# Osteochondromatosis - An uncommon cause of hemoptysis

**DOI:** 10.36416/1806-3756/e20250458

**Published:** 2026-03-05

**Authors:** Eduardo Just da Costa e Silva, Silvio Cavalcanti de Albuquerque, Edson Marchiori

**Affiliations:** 1. Instituto de Medicina Integral Professor Fernando Figueira - IMIP - Recife (PE) Brasil.; 2. Universidade Federal do Rio de Janeiro, Rio de Janeiro (RJ) Brasil.

**Figure 1:**
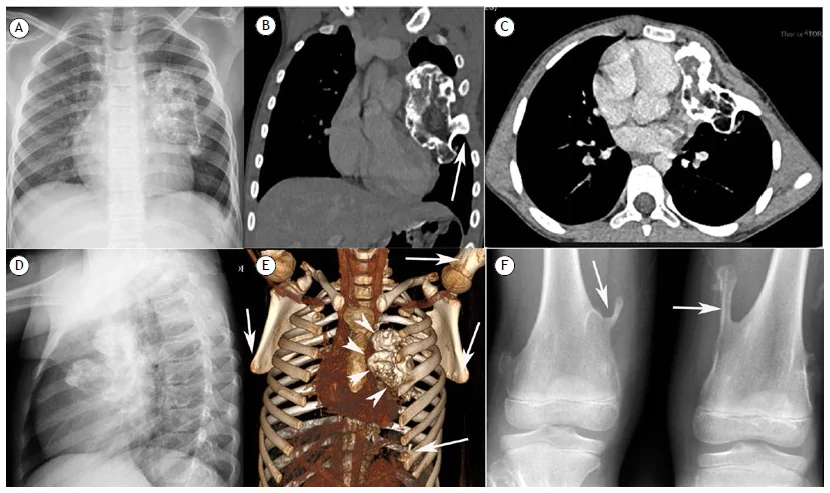
Frontal (A) and lateral (B) chest radiographs showing an irregular calcified mass in the left hemithorax. (C) Coronal reconstruction with mediastinal window setting demonstrated that the partially calcified mass originated mainly from the left fourth costal arch (arrow). (D) Three-dimensional coronally reconstructed image showing that the lesion was related to the third and fourth ribs (arrowheads). Also note exostoses in the scapulae, left humerus, and left seventh rib (arrows). (E) Axial section showing the relationship of the mass to mediastinal structures. (F) Radiograph of the knees showing pedunculated osteochondroma in both femurs (arrows).

A 7-year-old girl presented with nasal obstruction, rhinorrhea, and cough for 15 days. In the previous week, she had experienced two episodes of hemoptysis. Chest X-ray showed a “consolidation” in the left hemithorax (Figure 1A and 1B). She denied fever, headache, weight loss, chest pain, and respiratory distress. Chest CT showed a heterogeneous mass with irregular peripheral calcification originating from the left fourth rib. Small exostoses were also observed in other costal arches and in the scapulae, left humerus, and femurs, characteristic of hereditary multiple exostoses (multiple osteochondromatosis; Figure 1C-F). The hemoptysis was interpreted as probably resulting from the traumatic effect of the lesion on the lung parenchyma. A video-assisted thoracotomy was performed with resection of the pedunculated lesion and reconstruction of the chest wall. Histopathological examination of the specimen confirmed the diagnosis of osteochondroma. The patient had a good postoperative recovery.

Osteochondromas (exostoses) appear as single, isolated lesions or multiple lesions, as in hereditary multiple exostoses. Costal osteochondromas are rare, and they infrequently cause life-threatening thoracic injuries such as pneumothorax, hemothorax, and laceration of the lung due to perforation or repeated friction between the tumor and the visceral pleura. Surgical intervention is indicated for symptomatic lesions.^1^ ^-^ ^3^
